# Dendritic Architecture of Principal Basolateral Amygdala Neurons Changes Congruently with Endocrine Response to Stress

**DOI:** 10.3390/ijerph14070779

**Published:** 2017-07-14

**Authors:** Akshaya Hegde, Poh Soh Yee, Rupshi Mitra

**Affiliations:** School of Biological Sciences, Nanyang Technological University, 60 Nanyang Drive, Singapore 637551, Singapore; akshaya@resilieo.com (A.H.); joeypohsohyee@gmail.com (P.S.Y.)

**Keywords:** anxiety, corticosterone, inter-individual variation, amygdala, neuroendocrine, predator stress

## Abstract

Animals cope with changing environments through changes in behavior. Such plasticity is, however, marked by substantial inter-individual variability. Neuroendocrine reactivity to challenging environments can be an important predictor of resilience. Both basolateral amygdala (BLA) neurons and adrenal glucocorticoid signaling are integral parts of the stress neuroendocrine response. In this report, we test if individual variation in hormonal response to stress is associated with individual variation in the dendritic complexity of BLA neurons. We report a positive correlation between inter-individual variability in glucocorticoid response and neuronal plasticity in the BLA subsequent to a stressor. This suggests that stressful experiences in the past act as significant sculptors of BLA neuronal plasticity and congruent neuroendocrine response.

## 1. Introduction

Animals frequently respond to changes in the environment by changing their endocrine milieu. For example, episodic environmental challenges lead to a rise in adrenal hormones, including glucocorticoids [1]. This rise leads to the realignment of physiological resources towards more immediate needs of coping and survival, commonly known as a flight-or-fight response [2,3]. Different species show large variation in reactivity of endocrine change, or correspondence between environmental challenge and endocrine change [4,5]. Species endemic to predictable environments exhibit less reactivity, and species with more variable ecologies exhibit tighter coupling of environmental conditions with endocrine change [6,7,8]. Interestingly, individuals within the same population also exhibit remarkable variation in endocrine reactivity [4,5,9,10]. The coefficient of variation for glucocorticoid response to a threatening environment lies at 98% for the data presented in this report. Such large variation is consistent with previously published reports and meta-analyses conducted in a variety of animals. For example, meta-analytic estimates for the coefficient of variation at 30 to 60 min after restraint stress range from 50% to 60% in a variety of animals [4]. These numbers suggest that, generally, a large number of individuals exhibit significant departures from mean glucocorticoid response of the group. Biological underpinnings of such large inter-individual variation remain understudied at the present.

Exposure to traumatic and/or chronic stress often leads to increased anxiety-like behavior in rodents. The anxiogenic effect of prior stress has been confirmed in a variety of paradigms, including chronic restraint [11,12,13], acute immobilization [14], and exposure to predators [15,16]. Increased anxiety-like behavior in these experiments is long-lasting for weeks after termination of the stress. Stress also causes long-lasting neuroendocrine changes in parallel to sustained anxiogenesis. On one hand, historically stressful episodes potentiate the endocrine response to subsequent stressors [9,17,18]; while on the other hand, stress initiates structural plasticity in the basolateral amygdala (BLA). This plasticity is characterized by dendritic hypertrophy [19] and increase in spine density [20] of principal projection neurons within the BLA.

Several strands of evidence suggest that the effects of stress on anxiety-like behavior, BLA dendrites, and adrenal hormones are not isolated biological entities. Rather, these effects likely represent nodes of a reciprocally interacting loop. For example, exogenous corticosterone treatment after cat exposure leads to greater anxiety in rats when measured after several days [9]. This anxiogenesis manifests as a lower exploration of open spaces in elevated plus mazes and greater acoustic startle. This is congruent with the ability of exogenous corticosterone to increase anxiety-like behavior by itself, in parallel to causing dendritic hypertrophy in the BLA [21]. Similarly, pharmacological blockade of steroidogenesis in rats prevents a rise in anxiety-like behavior after exposure to cat odor [22]. Molecular studies also support the notion that the BLA is crucial for the relationship between glucocorticoids and stress-induced anxiogenesis. For example, diversion of glucocorticoid signaling within the BLA from glucocorticoid receptors toward mineralocorticoid receptors [23] or towards estrogen receptors [24] reduces anxiety-like behavior. Moreover, experimental manipulations that reduce BLA hypertrophy also show concomitantly reduced anxiety-like behavior and reduced glucocorticoid levels [25,26,27].

These observations suggest that BLA hypertrophy, anxiety and hypercortisolism are part of an interacting triad that is facilitated by historical stress. This hypothesis predicts a positive relationship of BLA dendrites with anxiety-like behavior and/or glucocorticoid levels. An alternative interpretation is also possible. The observations above could be similarly explained if BLA dendritic hypertrophy were a compensatory response to mediators of anxiety-like behavior and/or endocrine activation. This hypothesis predicts a negative relationship between BLA dendrites and anxiety-like behavior and/or hormonal measures. We test these competing predictions in the present report by quantifying correlations between endpoints related to anxiety-like behavior, BLA plasticity and glucocorticoids within the same cohort of individual rats. This experimental design also encapsulates inter-individual variation for glucocorticoid response to novel stressful episodes subsequent to historical stressors, and the relationship of this inter-individual variation with that of the BLA dendritic structure.

## 2. Materials and Methods

### 2.1. Animals and Experimental Groups

**Figure 1:**
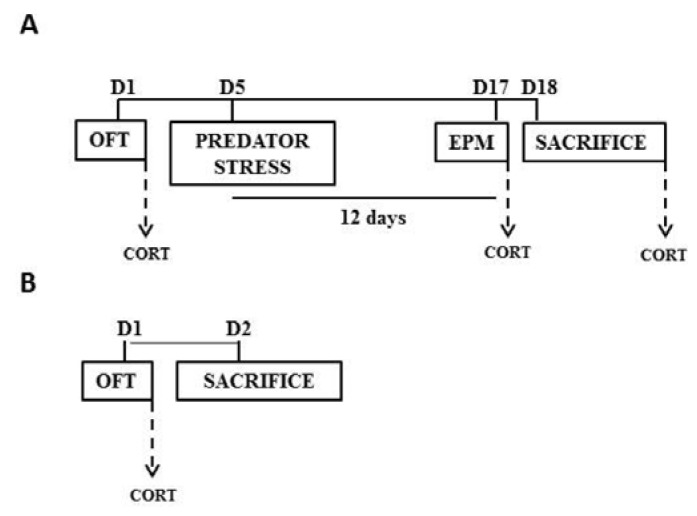
Schematic of experimental flow. (**A**) Animals were sequentially tested for anxiety in the open-field arena (OFT), exposed to predator stress and subsequently exposed to an elevated plus maze (EPM). Blood for corticosterone estimates was drawn thirty minutes after OFT, EPM, and the animals were eventually sacrificed for morphological endpoints. Days elapsed from the start of the experiment are denoted on top. (**B**) A separate cohort was exposed to open field and sacrificed the next day for morphological endpoints. Blood was collected 30 min after OFT for hormone measurement.

Adult male Wistar rats (7 weeks old, weight: 220–250 g, housed as 2 rats/cage; *ad-libitum* food and water; 12:12 light-dark cycles with lights on at 700 h) were procured from National University of Singapore. This outbred strain was chosen because of its greater phenotypic variability for later correlational analysis. Rats were habituated for one week before the start of the experiments. All experimental procedures were reviewed and approved by the institutional animal use and care committee of Nanyang Technological University (IACUC: A-0195). The experimental sequence is depicted in Figure 1.

### 2.2. Open Field Test (OFT)

Animals were tested for anxiety-like behavior in an open field. Exposure to open field occurred before exposure to the stressor, i.e., before exposure to cat odor (Figure 1A). In addition, a separate cohort of animals was exposed to OFT without subsequent stress exposures (Figure 1B). A square-shaped open field was constructed from Plexiglas (100 cm × 100 cm, 30 cm wall) and illuminated at the center (10 lux at center and 3–4 lux at the periphery). All tests were conducted between 900 and 1200 h (duration = 300 s). Time spent in the central part of the open field (33 cm × 33 cm) was measured as a proxy for lower anxiety-like behavior.

### 2.3. Predator Odor Exposure

Rats were exposed to 2 mL bobcat urine on day 5 of the experimental sequence (Figure 1A). The predator odor served as a stressor in our experiment [28,29,30,31]. Effect of predator odor stress on the subsequent reactivity of endocrine response and anxiety-like behavior was later measured in the elevated plus maze on day 17 Figure 1A. Exposure to predator odor occurred in a rectangular arena (two 76 cm × 9 cm bisects separated by a 9 cm × 9 cm central connector, wall = 15 cm; duration = 10 min). Individuals were habituated to the arena for 10 min on three successive days before actual exposure to the predator odor (bobcat urine). Aversion to the bobcat urine was quantified as occupancy of the bisect containing odor (76 cm × 9 cm) relative to the total area of the arena (chance = 47.2%, based on the area of the bisect containing predator odor vis-à-vis total area of the arena).

### 2.4. Elevated Plus Maze (EPM)

Animals were exposed to an EPM on day 17 of the experimental sequence (Figure 1A), between 900 and 1200 h, trial duration = 300 s). Relative open-arm exploration in the EPM was measured as a proxy for lower anxiety. The EPM consisted of two open (75 cm × 11 cm, illuminated <5 lux) and two enclosed (75 cm × 11 cm, non-illuminated) arms. The maze was elevated at a height of 60 cm from the ground.

### 2.5. Morphological Analysis of BLA Neurons

**Figure 2:**
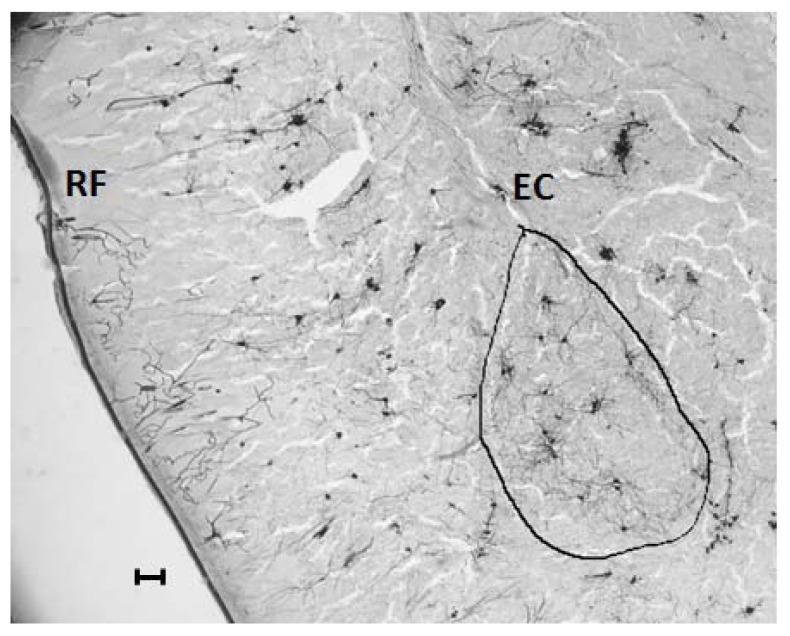
The region of interest sampled for dendritic arbors and spine density of the BLA neurons (bounded by black outline). RF denotes the location of the rhinal fissure, and EC denotes the location of the external capsule. The dorsal surface is towards the top of the figure and the medial surface is towards the right. Scale bar = 100 µm).

One day after EPM, brains were harvested after sacrifice by rapid decapitation (Figure 1A). Golgi staining was performed using commercially available reagents (FD Neurotechnologies, Columbia, MD, USA). Brain sections spanning the BLA between bregma of −2.04 and −3.36 were collected, counterstained with cresyl violet and cover-slipped in non-aqueous medium. Camera Lucida tracings of BLA neurons were produced using 400× magnification (Olympus B×43, Tokyo, Japan). Pyramidal or modified pyramidal spiny projection neurons of BLA were chosen [32] through random sampling within the BLA (Figure 2). Inclusion criteria included uniform impregnation of stain, un-truncated dendrites, and absence of visual interference from overlapping neurons. Tracings were digitized (8-bit grayscale TIFF, 300 dpi) for subsequent computerized image-analysis using custom-designed macros operating within ImageJ (http://rsb.info.nih.gov/ij/). Dendritic length and branch points were enumerated in eight neurons per animal, and the median of eight neurons was used for subsequent correlational analysis. A sampling of eight neurons per animal provided a stable estimate of median within each individual animal, as determined by the jackknife resampling model. Dendritic spines were manually counted at 1000× magnification, using oil-immersion objective lens. Dendrites directly originating from the cell soma were classified as primary dendrites, and those originating from primary dendrites were classified as secondary dendrites. Starting from the origin of the branch, and continuing away from the cell soma, spines were counted along a 60 µm stretch of the dendrite.

### 2.6. Corticosterone Estimation

Venous blood was collected via tail nicks at two points during the experiment (Figure 1A): before exposure to predator odor (i.e., 30 min post-OFT), and after EPM (i.e., 30 min post-EPM), subsequent to exposure to predator odor. Additionally, trunk blood was collected at the point of sacrifice. A separate cohort was used to collect blood at 30 min post-OFT without subsequent exposure to predator odor or EPM (Figure 1B). The concentration of serum corticosterone was quantified using a commercial enzyme-linked immunoassay (Assay Designs; Enzo^®^ Life Sciences, Farmingdale, NY, USA).

### 2.7. Statistical Analysis

Spearman’s rank correlation coefficients were calculated for pairs of experimental endpoints. A *p* value ≤ 0.05 was deemed as the cut-off for statistically significant correlations. N is listed in Table 1 and Table 2. Additionally, principal component analysis was performed to delineate patterns of correlation in the observed dataset. Software IBM SPSS Statistics v23 (IBM, Armonk, NY, USA) was used for all statistical analysis.

## 3. Results

**Figure 3:**
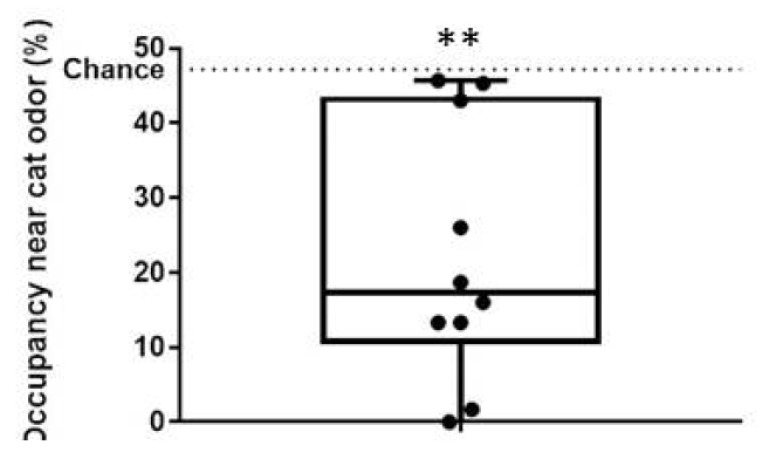
Aversion to bobcat urine in male rats used during the experiment. Ordinate depicts occupancy in the cat odor bisect of the arena, and the dashed line represents theoretical expectancy based on chance alone. Each dot represents the raw data from one male rat. Box plot depicts median, 25th percentile, and 75th percentile. ** *p* < 0.005; one-sample t-test against the chance of 47.2%.

Ten male rats were tested for pre-stress anxiety-like behavior in OFT, exposed to predator odor stress, and tested for post-stress anxiety-like behavior in EPM (Figure 1A). Congruent with previous studies [28,29,30,31], rats exhibited significant aversion to bobcat odor (Figure 3; one-sample *t*-test with chance level of 47.2%, t_9_ = −4.58, *p* = 0.001). Out of ten animals, post-stress anxiety-like behavior in EPM could not be measured in one animal, and spine density of BLA neurons could not be measured in two animals, due to procedural failures. The sample size available for subsequent analysis is listed in Table 1. In addition, Table 1 depicts sample statistics for all endpoints in these animals, including estimates of mean, standard error of mean, median, and range. Aversion to predator odor did not exhibit statistically significant correlations with corticosterone levels post-EPM (*p* = 0.80), median number of BLA branch points (*p* = 0.72), median spine density on primary BLA dendrites (*p* = 0.18), percentage open arm time in EPM (*p* = 0.67), or center time in open field (*p* = 0.50).

**Figure 4:**
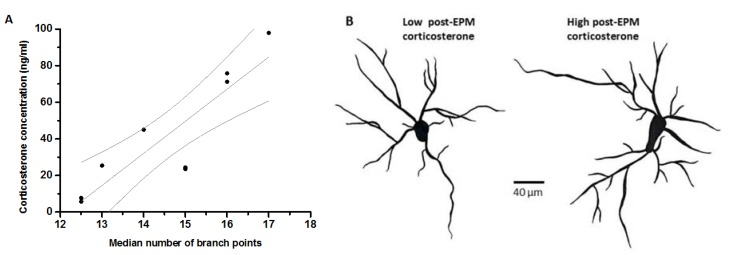
Correlation between branch points and corticosterone concentration. The abscissa of panel (**A**) depicts number of branch points per basolateral amygdala neuron (median from a sample of eight neurons sampled per animal). Ordinate depicts serum corticosterone concentration (ng/mL) thirty minutes after exposure to the elevated plus-maze. Best linear fit is also depicted with 95% confidence interval (R^2^ = 0.75, slope = 17.5 ± 3.5; n = 9 animals; grey lines). Panel (**B**) depicts representative BLA neuronal tracings from animals exhibiting low (**left**, 5.7 ng/mL) and high (**right**, 71.2 ng/mL) corticosterone response post-EPM. Scale bar = 40 µm.

**Figure 5:**
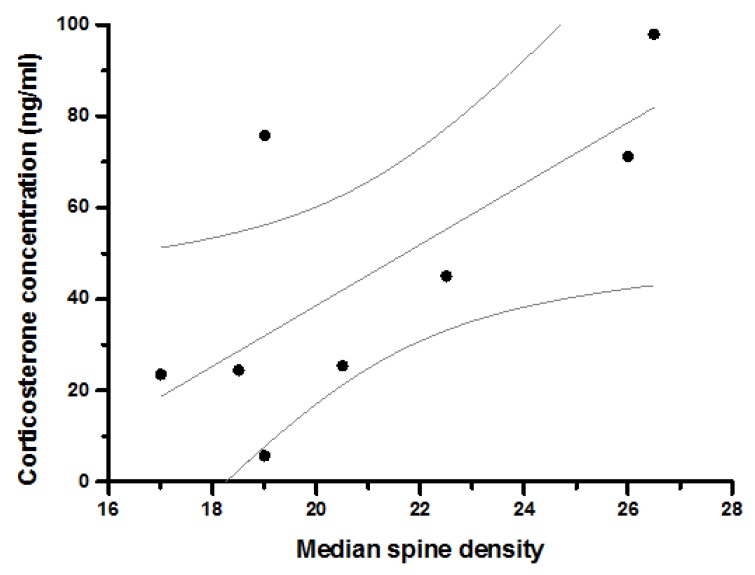
Correlation between spine density and corticosterone concentration. Abscissa depicts density of spines on primary shaft of basolateral amygdala neurons (median from a sample of eight neurons per animal; per 60 µm). Ordinate depicts serum corticosterone concentration (ng/mL) thirty minutes after exposure to the elevated plus-maze. Best linear fit is also depicted with 95% confidence interval (R^2^ = 0.46, slope = 6.6 ± 2.5; n = 8 animals; grey lines).

A correlation matrix was calculated for pair-wise comparisons of endpoints, pertaining to anxiety-like behavior, BLA structure and corticosterone concentration using Spearman’s procedure (Table 2). Concentration of plasma corticosterone thirty minutes post-EPM was positively correlated with the number of branch points of BLA neurons (Figure 4A; *rho* = 0.638, *p* ≤ 0.05). We further performed a linear regression between these two endpoints. Values for seven out of nine animals were constrained within the 95% confidence limit of the best linear fit (Figure 4A). Representative neurons from animals showing high and low corticosterone response post-EPM are depicted in Figure 4B. Similarly, corticosterone concentration post-EPM was also positively correlated with the number of spines on the primary shaft of BLA neurons (Figure 5; *rho* = 0.719, *p* ≤ 0.05). Values for six out of eight animals were constrained within 95% confidence limit of the best linear fit.

Corticosterone concentration post-EPM was positively correlated with trunk blood corticosterone obtained at the point of sacrifice. We did not observe a statistically significant correlation between corticosterone concentration post-OFT and post-EPM; collection points before and after intervening predator odor exposure (Table 2; *rho* = −0.079, *p* = 0.83, β or probability of type 2 error at accepted α of 0.05 = 0.044). Similarly, open arm exploration in EPM was not significantly correlated with either corticosterone concentration (Table 2; *rho* = 0.633 and −0.008, *p* = 0.067 and 0.98, for % entries and time in open arm, respectively) or architecture of BLA neurons (*rho* = 0.097 to 0.360, *p* = 0.43 to 0.80). Correlation between BLA dendritic endpoints and corticosterone concentration post-OFT (i.e., before cat odor exposure) also did not reach statistical significance.

**Figure 6:**
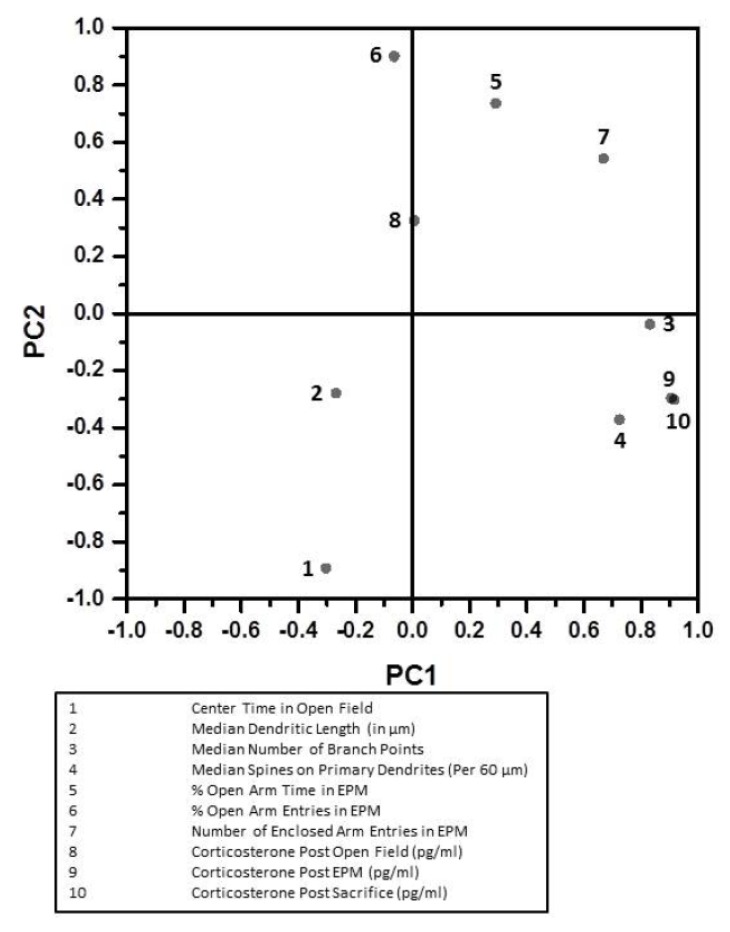
Principal component analysis of the measured endpoints. Initial two dimensions are depicted along abscissa and ordinate. Numerals adjacent to dots represent the endpoints, listed in the inset underneath.

Principal component analysis was further used to orthogonally transform quantified endpoints. Ten endpoints related to anxiety-like behavior, BLA structure and corticosterone concentration (Figure 6) were used for dimension reduction. The principal axis method was utilized to extract the components. Only the first two principal components displayed eigenvalues greater than 2. These two principal components extracted 65.8% of the total variance in the dataset (36.3% and 29.6% of total variance, respectively). An endpoint was determined to load on a given component if the factor loading exceeded 0.5 for that component and was less than 0.5 for the alternative component. Using these criteria, four endpoints were found to load on the first principal component. These were post-EPM corticosterone concentration (0.913), post-sacrifice corticosterone concentration (0.923), the number of branch points on the BLA neurons (0.837), and spine density of the BLA neurons (0.731). On the other hand, three endpoints were found to load on the second principal component. These were time spent in the center of the open field (−0.901), percentage of time spent in the open arm of the EPM (0.732), and percentage of entries into the open arms of the EPM (0.897). Figure 6 depicts endpoints with references to first two principal components. When plotted this way, post-EPM and post-sacrifice corticosterone concentration loaded congruently with BLA branches and spines (Figure 6, lower right quadrant).

A separate cohort of control male rats was exposed to a novel open field arena, followed by measurement of corticosterone and BLA dendritic measurement without intervening exposure to predator odor (Figure 1B). The concentration of plasma corticosterone post-OFT in this cohort did not exhibit significant correlation with dendritic length or number of branch points of BLA neurons (n = 6 animals, *rho* = −0.086, *p* = 0.92). Additionally, we did not observe a significant correlation between plasma corticosterone post-OFT and spine density on primary dendrites of BLA neurons (*rho* = −0.486, *p* = 0.36). Time spent in the anxiogenic center of the open field was not correlated with any of the dendritic measurements in the BLA (*p* > 0.17).

## 4. Discussion

In this report, we present correlative evidence that inter-individual variation in glucocorticoid levels co-elutes with variation in BLA dendritic arbors. Individuals with more complex arbors and more dendritic spines also exhibit greater post-stress levels of circulating glucocorticoids after exposure to the elevated plus-maze. This positive relationship supports the notion that BLA and glucocorticoids represent two interacting nodes of neuroendocrine plasticity in response to historical stress. In addition to positive correlation, this possibility is also supported by congruent loading of hormone levels with BLA dendritic measure during principal component analysis. Several studies have reported that experimental manipulation of BLA dendritic arbors causes a systematic change in circulating glucocorticoids. Inverse manipulation of glucocorticoids also causes systematic changes in BLA dendritic arbors. Experimental reduction in BLA activity through overexpression of Ca^2+^-activated K^+^ channels results in reduction in BLA arbors and lower glucocorticoid levels post-stress [25]. Chronic infection with a protozoan parasite *Toxoplasma gondii* causes dendritic atrophy in BLA and reduction in circulating glucocorticoids [27]. Polysynaptic pathways facilitate influence of the amygdala on stress endocrine axis [33]. These pathways include efferents of BLA to other brain regions, such as the central amygdala, medial amygdala, and bed nucleus of stria terminalis, which then project to the paraventricular nucleus of the hypothalamus, tapping into the stress endocrine axis. BLA neurons show activation in response to restraint stress or footshock, demonstrating that psychogenic stressors can initiate BLA activity [34,35,36]. On the other hand, subcutaneously supplied exogenous corticosterone causes dendritic expansion in BLA [21] similar to that observed due to stress [19,20]. Disruption of glucocorticoid binding within BLA blunts behaviors that are dependent on amygdala activity like anxiety [24] and fear conditioning [37,38]. Similarly, competitive reduction of glucocorticoid receptor binding through over-expression of mineralocorticoid receptors within BLA reduces post-stress glucocorticoid release [23]. Thus, effects of glucocorticoids on BLA arbors and effects of arbors on glucocorticoid levels are likely reciprocal parts of a syndromic and plastic response to the stress. The present study takes an inter-individual, rather than inter-group, approach to the interaction of BLA arbors and stress endocrine axis. We show that the positive relationship between these nodes remains consistent for individuals across a wide range of stress sensitivity.

Previous studies, detailed above, have established that experimental manipulations that change glucocorticoid levels also change BLA dendritic arbors. For example, stress or exogenous glucocorticoids both cause BLA dendritic expansion [13,19,21]. This is also supported by the observation that prenatal stress significantly increases the volume of the lateral amygdala [39] in parallel to increasing glucocorticoid levels and causing adrenal hypertrophy [40]. It remains unclear if structural plasticity in these cases represents a compensatory change, or reflects facilitation of interaction between BLA and stress hormones. Dendritic expansion can reduce input resistance of neurons, thereby increasing quiescence in reciprocal interaction with the endocrine axis. Alternatively, the addition of synaptic inputs on dendritic cables can increase the magnitude of synaptic currents, thereby facilitating the interaction. Experimental manipulations with concurrent changes in structure and hormones cannot dissect these competing possibilities. The positive correlation and congruent loading for structural and endocrine endpoints reported here argue that BLA changes do not reflect a compensatory response.

The relationship between BLA neurons and emotional resilience has been previously studied [41,42]. However, both of these reports studied rats at two extremes of anxiety-like behavior response several days after exposure to a live cat. Individual rats with greater anxiety-like behavior also exhibited greater dendritic complexity in these studies. The present study complements these studies through its focus on inter-individual variation along the complete parametric space available in the animal model. Our experiment also used cat urine, rather than a live cat, as the initial stressor. Cat urine is a partial predator cue which is less predictive of the cat presence and hence is less stressful [43]. Our goal in choosing this stimulus was to increase inter-individual variability available for correlational analysis.

It should be noted that the current evidence is mainly correlative in nature. For example, it can be argued that inter-individual variability in the stress endocrine axis results in variable effects of predator odor on BLA dendritic arbors. From this perspective, BLA structural plasticity and post-stress glucocorticoids can be viewed as two independent manifestations without a functional relationship. Such independence is unlikely in view of prior work showing congruence between BLA plasticity and future endocrine response during experimental manipulations. It is more parsimonious to assume that dendritic changes in the BLA and changes in post-EPM glucocorticoid levels reflect a reciprocal and functional relationship that encapsulates the effects of historical experience. Yet an unequivocal demonstration will require investigation across a wider range of phenotypic diversity, e.g., using a hybrid mouse diversity panel or collaborative crosses. We hope that the present observations will provide an impetus to these experiments in future. Conclusions drawn in this study are limited by the relatively low sample size. This may have precluded discovery of other associations that were biologically present but could not be discerned from the available statistical power of the study. This is particularly relevant to the association between dendritic arbors of the BLA and anxiety-like behavior measured in the EPM, in view of previous reports that stress concomitantly causes anxiogenesis and BLA dendritic expansion [12,19,21]. It can also be argued that association between glucocorticoid response to the EPM and the BLA structure reflects a consequence of behavioral testing rather than historical stressor of predator odor. Time elapsed between the quantification of anxiety-like behavior in plus-maze and sacrifice was short. It is unlikely that the stress of the elevated plus-maze itself resulted in structural plasticity in the BLA because these changes are known to require a chronic time-frame [20]. Nevertheless, we do not unequivocally demonstrate a lack of association between structural and endocrine endpoints in the absence of predator odor exposure.

BLA dendritic changes, especially branching of the arbors, occur over a relatively prolonged timeframe. For example, dendritic changes in the BLA due to restraint stress after a single session of acute restraint requires a chronic window of ten days to manifest and remain unobservable one day after the stress exposure [20]. Similarly, acute treatment with extraneous corticosterone leads to dendritic hypertrophy in the BLA [21]. In our present study, twenty-four hours elapsed between EPM exposure and sacrifice of the animals. Thus, it is restrictive to assume that the greater BLA dendritic complexity was associated with greater corticosterone levels after EPM, rather than greater post-EPM stress hormones leading to more complex BLA dendrites.

## 5. Conclusions

In conclusion, we report that BLA dendritic architecture varies congruently with stress hormone response to a novel stressor after exposure to predator odor. This suggests that BLA neurons are important mediators of inter-individual variation in stress-sensitivity and endocrine response to the challenging environment.

## Figures and Tables

**Table 1:** Sample statistics for endpoints measured in this study.

Estimates	Center Time in Open Field	Median Dendritic Length (in Microns)	Median Number of Branch Points	Median Spines on Primary Dendrites (per 60 Microns)	% Open Arm Time in EPM	% Open Arm Entries in EPM	Number of Enclosed Arm Entries in EPM	Corticosterone Post Open Field (pg/mL)	Corticosterone Post EPM (pg/mL)	Corticosterone Post Sacrifice (pg/mL)
Median	3.50	1529	14.5	19.75	7.33	27.27	7	14.10	35.20	48.50
Range	12	499	4.5	9.50	40.33	46.67	7	38.40	194.10	151.10
Mean	4.90	1581	14.5	21.13	14.52	24.86	6	18.09	57.64	62.38
SEM	1.269	55	0.5	1.25	4.77	4.76	1	4.17	18.56	14.81
N	10	10	10	8	9	9	9	10	10	10

**Table 2:** Spearman’s rank correlation coefficients for endpoint pairs. The number of animals and *p* values are also listed. Shaded cells depict endpoint pairs with statistically significant correlation.

		Median Dendritic Length (in Microns)	Median Number of Branch Points	Median Spines on Primary Dendrites (per 60 Microns)	% Open Arm Time in EPM	% Open Arm Entries in EPM	Number of Enclosed Arm Entries in EPM	Corticosterone Post Open Field (pg/mL)	Corticosterone Post EPM (pg/mL)	Corticosterone Post Sacrifice (pg/mL)
**Center Time in Open Field**	Coefficient	0.216	0.013	0.216	−0.616	−0.809	−0.562	−0.370	−0.191	0.179
	Sig.	0.549	0.973	0.607	0.077	0.008	0.115	0.292	0.596	0.621
	N	10	10	8	9	9	9	10	10	10
**Median Dendritic Length (in microns)**	Coefficient		−0.067	0.623	−0.200	−0.017	−0.280	0.418	−0.224	−0.321
	Sig.		0.853	0.099	0.606	0.966	0.466	0.229	0.533	0.365
	N		10	8	9	9	9	10	10	10
**Median Number of Branch Points**	Coefficient			0.370	0.101	0.097	0.185	0.301	0.638	0.190
	Sig.			0.367	0.795	0.803	0.635	0.399	0.047	0.599
	N			8	9	9	9	10	10	10
**Median Spines on Primary Dendrites (per 60 microns)**	Coefficient				0.360	−0.306	0.248	0.108	0.719	0.503
	Sig.				0.427	0.504	0.592	0.799	0.045	0.204
	N				7	7	7	8	8	8
**% Open Arm Time in EPM**	Coefficient					0.460	0.856	0.133	0.633	0.483
	Sig.					0.213	0.003	0.732	0.067	0.187
	N					9	9	9	9	9
**% Open Arm Entries in EPM**	Coefficient						0.460	0.477	−0.008	−0.351
	Sig.						0.213	0.194	0.983	0.354
	N						9	9	9	9
**Number of Enclosed Arm Entries in EPM**	Coefficient							−0.068	0.525	0.475
	Sig.							0.862	0.146	0.197
	N							9	9	9
**Corticosterone Post Open Field (pg/mL)**	Coefficient								−0.079	−0.418
	Sig.								0.829	0.229
	N								10	10
**Corticosterone Post EPM (pg/mL)**	Coefficient									0.685
	Sig.									0.029
	N									10
